# Substrate-integrated photonic doping for near-zero-index devices

**DOI:** 10.1038/s41467-019-12083-y

**Published:** 2019-09-11

**Authors:** Ziheng Zhou, Yue Li, Hao Li, Wangyu Sun, Iñigo Liberal, Nader Engheta

**Affiliations:** 1 Department of Electronic Engineering, Tsinghua University, 100084 Beijing, China; 2 Department of Electrical and Electronic Engineering, Public University of Navarre, Pamplona, 31006 Spain; 3 Multispectral Biosensing Group, Navarrabiomed, Irunlarrea 3, Pamplona, 31008 Navarra Spain; 4 Department of Electrical and Systems Engineering, University of Pennsylvania, Philadelphia, PA 19104 USA

**Keywords:** Electrical and electronic engineering, Materials for devices, Metamaterials

## Abstract

Near-zero-index (NZI) media, a medium with near zero permittivity and/or permeability, exhibits unique wave phenomena and exciting potential for multiple applications. However, previous proof-of-concept realizations of NZI media based on bulky and expensive platforms are not easily compatible with low-cost and miniaturization demands. Here, we propose the method of substrate-integrated (SI) photonic doping, enabling the implementation of NZI media within a printed circuit board (PCB) integrated design. Additionally, the profile of the NZI device is reduced by half by using symmetries. We validate the concept experimentally by demonstrating NZI supercoupling in straight and curve substrate integrated waveguides, also validating properties of position-independent photonic doping, zero-phase advance and finite group delay. Based on this platform, we propose design of three NZI devices: a high-sensitivity dielectric sensor, an efficient acousto-microwave modulator, and an arbitrarily-curved ‘electric fiber’. Our results represent an important step forward in the development of NZI technologies for microwave/terahertz applications.

## Abstract (web summary)

Here, the authors demonstrate substrate-integrated photonic doping, enabling the implementation of near-zero-index media within a printed circuit board integrated design. They illustrate the potential by designing and numerically demonstrating a dielectric sensor, an acousto-microwave modulator and a flexible transmission line.

## Introduction

Materials with unconventional electromagnetic properties are of great interest in both fundamental research and modern engineering applications. Traditional plasmonic materials^1–3^ and metamaterials^4–6^ have led to important theoretical advances, novel phenomena and applications, including negative refraction^7^, superresolution imaging^5,8^, cloaking^9–11^, ultra-small optical cavities^12^, and computing materials^13^, just to name a few. In recent years, the category of artificial materials exhibiting a near-zero refractive index (i.e., NZI media)^14,15^ have attracted much interest due to their unique wave phenomena. Different classes of NZI media include epsilon-near-zero (ENZ)^16–18^, mu-near-zero (MNZ)^19^, and epsilon-and-mu-near-zero (EMNZ)^20,21^ media, depending on which material’s constitutive parameter (permittivity, permeability, or both) approaches to zero. What all these media have in common is that a near-zero refractive index results in an effectively enlarged wavelength, and very large phase velocity for steady-state continuous-wave (CW) signals. As a result, fields within NZI media oscillate in unison, leading to spatially static field distributions even at microwave, infrared, and optical frequencies^21,22^. Based on this exceptional wave behavior, different functionalities of NZI media have been proposed, including tunneling electromagnetic energy through channels of arbitrary geometry^16,17^ (i.e., supercoupling), boosting nonlinear effects^23,24^, shaping radiation patterns^25,26^, geometry-invariant resonant cavities^27^, and modulating the individual and collective emission properties of quantum emitters^28,29^. However, the current challenge is to develop the technological platforms that can empower practical applications for these intriguing theoretical concepts, so as to enable NZI technologies^30^.

As with other exotic electromagnetic media, one of the main challenges in implementing these theoretical concepts is the availability of platforms to easily realize NZI media, which must be compatible with current technological demands. Experimental demonstrations of NZI media typically involve metals, semiconductors, or polar dielectrics operating around their plasma frequencies^31^. However, these methods, which might suffer from relatively high losses, can only provide an ENZ response. Metamaterials^32,33^ and photonic crystals^34–36^ have also been employed to realize different classes of NZI media by using periodic structures, but these may exhibit spatial dispersion, prohibitive fabrication costs and complexity in their designs. EMNZ-related resonant transmission effects in extended unit-cell transmission lines have been demonstrated by introducing periodically-loaded shunt inductors into a MNZ transmission line^37^. However, these are 1D systems based on lumped elements that may limit the geometry flexibility of the tunneling effect, and are challenging to scale to higher frequency ranges. Recently, the concept of photonic doping^38^ has expanded the possibility of realizing different NZI media. In this scheme, an ENZ medium, which can be emulated, e.g., by a waveguide at cutoff frequency^39^, serves as the background, while dielectric inclusions named as “photonic dopants” are employed to tune its internal and external fields. It is demonstrated that the impact of these dopants is the modification of the effective permeability, while maintaining an ENZ response. In doing so, photonic doping grants access to extreme material responses, such as EMNZ and perfect magnetic conductor (PMC) media. Remarkably, this effective material description goes beyond the usual regime of effective medium theories, and it is valid independently of the number, size, and position of the particles, as well as the shape of its ENZ host.

Photonic doping techniques have already been utilized to experimentally demonstrate EMNZ tunneling (i.e., enhanced transmission with zero-phase advance, independent on the deformation of the waveguide). However, this first proof-of-concept experiment was based on a bulky and high-profile rectangular waveguide, and using a very complex system for the assembly of the dopant particle. These characteristics have made it challenging to bring this phenomenon to most applications that demand miniaturization, low profile, low cost, and compatibility with integrated circuit architectures.

Here we show how the the concept of substrate-integrated (SI) photonic doping can be used to addressed these practical challenges. We aim to accommodate the original photonic doping concept in a planar architecture, the substrate-integrated waveguide^40^ (SIW), in which we implement a “doped” ENZ cavity simulating EMNZ media. The SIW shows its superiority over the rectangular waveguide for the easy assembly and high integration level with other electronic circuits. Moreover, we make use of different symmetries to improve the original photonic doping design, reducing the device cross-section to half its original size. We develop the theory to facilitate the design procedure, and experimentally demonstrate EMNZ tunneling in straight and curved configurations. The experimental results validate the position-independent, zero-phase progress and large group delay characteristics of EMNZ tunneling. We expect that the proposed platform will also open new opportunities for implementation of NZI technologies and, in particular, EMNZ media. We illustrate this potential by designing and numerically demonstrating three devices: (i) A dielectric sensor that takes advantage of the local field enhancement within and near the dielectric dopants. (ii) An acousto-microwave modulator that efficiently couples the output wave to deeply subwavelength mechanical movements. (iii) A flexible transmission line, we named the “electric fiber”, which harness geometry-invariant EMNZ tunneling to transfer signals through arbitrarily-curved routes in integrated circuits. We envision that the proposed SI photonic doping scheme may enrich the theory of non-periodic metamaterials and bring the exotic properties of NZI media into different fields, such as microwave engineering, optics, terahertz technology, communications engineering, and material science and engineering.

## Results

### Concept and theoretical analysis

**Fig. 1:**
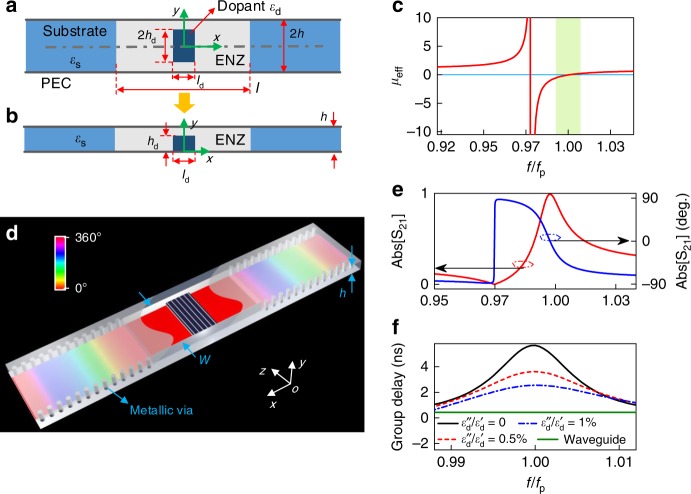
Evolution of the substrate-integrated (SI) photonic doping concept and its transmission properties**. a** 2D photonic doping structure. The ENZ cavity, which is empty and contains only a dielectric rod, is connected to the external environment with via waveguides filled with a material with *ε*_s_ = 2.65 and illuminated by a wave with the magnetic field polarized along the out-of-plane (*z*) axis. The origin of the coordinate is at the center of the dopant, and the geometric center of the dopant is aligned on the symmetry plane (dashed line) of photonic doping structure. The relative permittivity *ε*_d_ of dopant is 37. Geometry parameters are chosen as follows: *l* = 80 mm, *h* = 5 mm, *l*_d_ = 12 mm, and *h*_d_ = 2.4 mm. **b** SI photonic doping structure, the dopant of SI photonic doping is placed on the bottom PEC plate. **c** The calculated effective permeability. The frequency is normalized to *f*_p_ = 5.8 GHz. **d** Three-dimensional SI photonic doping structure implemented by substrate-integrated waveguide (SIW). The phase distribution of the wave at EMNZ supercoupling is schematically presented over the structure. The width *W* of the ENZ cavity is 25.8 mm. Calculated transmission coefficient (phase and amplitude), and group delay versus the loss of dopant for 3D SI photonic doping are presented in **e**, **f**. The group delay of the same-sized waveguide (width = 25.8 mm and length = 80 mm) with filled dielectric *ε*_s_ = 2.65 is calculated for comparison

The evolution of the SI photonic doping from the original photonic doping scheme is illustrated in Fig. 1. For simplicity, we start from the 2D photonic doping structure shown in the Fig. 1a, where the ENZ medium is doped with a dielectric impurity and illuminated by a wave with the magnetic field parallel with the out-of-plane (*z*) axis, and the electric field in the *x*–*y* plane. The origin of the coordinate system is assigned as shown in Fig. 1a. The new points of the proposed SI photonic doping evolved from the original photonic doping are summarized as follows: first, we use a rectangular dopant whose geometry adapts better to planar integrated electronic or optical circuits. Second, we use symmetries to reduce the volume of the device to half its original size. Third, the concept of substrate integration is introduced into the conventional photonic doping for building a new feasible way to realize and exploit the interesting features of near-zero-index materials in a low-cost and compact platform. Finally, we establish a complete theoretical framework to quantitatively and systematically investigate the property of the EMNZ resonance, such as group delay and field concentration.

Next, we provide the theoretical analysis of this configuration. To this end, we start by analyzing the response of a rectangular 2D dielectric dopant in Fig. 1a. In this 2D configuration, the magnetic field within the dopant satisfies the following wave equation and boundary condition:

1 $$\nabla ^2\psi + \varepsilon _{\mathrm{d}}(\omega /c)^2\psi = 0,\quad \left. \psi \right|_{\partial A_{\mathrm{d}}} = 1,$$

where *ε*_d_ is the relative permittivity of the dielectric dopant, *ω* is the angular frequency, and *c* is the vacuum speed of light. *A*_d_ represents the dopant region with size of 2 *h*_d_ × *l*_d_, whose boundary is denoted by $\partial A_d$. The boundary condition is imposed from the uniform magnetic field in the ENZ medium. We solve the Eq. (1) by Green’s function technique (detailed information can be found in Supplementary Note 1), and we find that the normalized magnetic field within the dopant can be written as follows:

2 $$\psi (x,y) = 1 + \mathop {\sum}\limits_{m = 1,n = 1}^{ + \infty } \varepsilon _{\mathrm{d}}\frac{{(2\pi f)^2}}{{c^2}}\frac{{4(( - 1)^m - 1)(( - 1)^n - 1)}}{{\pi ^2mn}}\\ \frac{{{\mathrm{cos}}(m\pi x/l_{\mathrm{d}}){\mathrm{cos}}(n\pi y/(2h_{\mathrm{d}}))}}{{\left( {m\pi /l_{\mathrm{d}}} \right)^2 + \left( {n\pi /(2h_{\mathrm{d}})} \right)^2 - \varepsilon _{\mathrm{d}}(2\pi f/c)^2}}$$

We note that the tangential electric field on the symmetry plane (shown in Fig. 1a as dashed gray line), is calculated by the directional derivative of *ψ* along the normal direction (*y*-axis): *E*_*τ*_ = (−*iωε*_0_*ε*_d_)^−1^[∂*ψ*(*x*, 0)/∂*y*] (Time convention *e*^*-iωt*^ is assumed). It can be readily checked that the value of the tangential *E* field is exactly equal to zero for the magnetic field distribution given by Eq. (2). This implies that the image principle can be applied to reduce the profile of the device. That is, grounding the classic photonic doping structure at the symmetry plane, we can achieve the same response by only half of volume of the original device, as shown in Fig. 1b. Once the field distribution within the dopant is known, the dispersive effective relative permeability of the doped ENZ cavity in the SI photonic doping structure can be calculated as follows^38^:

3 $$\mu (f) = 1 + \frac{1}{A}\left( {\mathop {\iint}\nolimits_{A_{\mathrm{d}}} \psi dA - A_{\mathrm{d}}} \right),$$

where *A* is the cross-section area of the ENZ cavity, given by 2*h* × *l* in our scenario, and *ψ* is the normalized magnetic field in Eq. (2). By substituting Eq. (2) into Eq. (3), we can show that:

4 $$\mu (f) = {\mathrm{1 + }}\mathop {\sum}\limits_{m = 1,n = 1}^{ + \infty } {\frac{{{\mathrm{4}}l_{\mathrm{d}}h_{\mathrm{d}}(( - 1)^m - 1)^{\mathrm{2}}(( - 1)^n - 1)^{\mathrm{2}}}}{{lh\pi ^4m^2n^2}}}\\ \frac{{\varepsilon _{\mathrm{d}}\left( {{\mathrm{2}}\pi f/c} \right)^2}}{{\left( {m\pi /l_{\mathrm{d}}} \right)^2 + \left( {n\pi /(2h_{\mathrm{d}})} \right)^2 - \varepsilon _{\mathrm{d}}\left( {{\mathrm{2}}\pi f/c} \right)^2}}$$

We set the parameters as *h* = 5 mm, *l* = 80 mm, *l*_d_ = 12 mm, *h*_d_ = 2.4 mm, and *ε*_d_ = 37, so that the first zero of Eq. (4) is calculated to be *f*_p_ = 5.8 GHz, and we plot the effective permeability as a function of frequency in Fig. 1c. The figure shows that the dispersion properties of the effective permeability are characterized by a clear resonant behavior, with a band of negative values above resonance, and a zero-crossing around the prescribed frequency *f*_p_. The length of the ENZ channel *l* also plays a role in tuning the zeros of effective permeability curve, with a reduced *l* contributing to a higher EMNZ frequency (detailed information can be found in Supplementary Note 2).

Up to this point, we have assumed an ideal 2D ENZ medium. Next, the ENZ medium is practically emulated by an air-filled 3D waveguide operating around the cutoff frequency of TE_10_ mode. The width of the waveguide *W* is set as 25.8 mm, so that the cutoff frequency (=0.5*c*/*W*) is equal to *f*_p_ = 5.8 GHz. The doped ENZ cavity is therefore equivalent to a slab with permeability *μ*(*f*) and permittivity *ε*_enz_ = 1−(*f*_p_/*f*)^2^, which is described by the following transfer matrix^41^:

5 $$A_{\mathrm{C}} = \left[ {\begin{array}{*{20}{c}} {{\mathrm{cos}}\left( {\sqrt {\varepsilon _{{\mathrm{enz}}}\mu (f)} \frac{\omega }{c}l} \right)} & { - i\eta _0\sqrt {\frac{{\mu (f)}}{{\varepsilon _{{\mathrm{enz}}}}}} {\mathrm{sin}}\left( {\sqrt {\varepsilon _{{\mathrm{enz}}}\mu (f)} \frac{\omega }{c}l} \right)} \\ {\frac{{ - i}}{{\eta _0}}\sqrt {\frac{{\varepsilon _{{\mathrm{enz}}}}}{{\mu (f)}}} {\mathrm{sin}}\left( {\sqrt {\varepsilon _{{\mathrm{enz}}}\mu (f)} \frac{\omega }{c}l} \right)} & {{\mathrm{cos}}\left( {\sqrt {\varepsilon _{{\mathrm{enz}}}\mu (f)} \frac{\omega }{c}l} \right)} \end{array}} \right],$$

where *η*_0_ = 377 Ohm is the intrinsic wave impedance in vacuum. To integrate the structure of photonic doping with the planar circuits, the SIW is introduced to feed the ENZ cavity. By shorting the upper and lower metallic plates with metallic vias through the substrate, the SIW can support the modes of the traditional rectangular waveguide but features the easy assembly and excellent compatibility with planar circuits. Finally, the typical architecture of the SI photonic doping with the predicted phase distribution for magnetic field at EMNZ resonance is presented in Fig.1d. After being connected with the feeding SIW, the total transfer matrix of the system is given by:

6 $$A = \left[ {\begin{array}{*{20}{c}} {\left( {\eta _0/\sqrt {\varepsilon _{\mathrm{f}}} } \right)^{ - \frac{1}{2}}} & 0 \\ 0 & {\left( {\eta _0/\sqrt {\varepsilon _{\mathrm{f}}} } \right)^{\frac{1}{2}}} \end{array}} \right]A_C\left[ {\begin{array}{*{20}{c}} {\left( {\eta _0/\sqrt {\varepsilon _{\mathrm{f}}} } \right)^{\frac{1}{2}}} & 0 \\ 0 & {\left( {\eta _0/\sqrt {\varepsilon _{\mathrm{f}}} } \right)^{ - \frac{1}{2}}} \end{array}} \right],$$

where *ε*_f_ = *ε*_s_ − (*f*_p_/*f*)^2^ is the effective relative permittivity of the SIW. Through the transformation from transfer matrix to scattering matrix (see Methods for detailed information) the transmission coefficient is then given by:

7 $$\begin{array}{*{20}{l}} {S{}_{21}(f)} \hfill & = \hfill & {\frac{2}{{A_{11} + A_{12} + A_{21} + A_{22}}}} \hfill \\ {} \hfill & = \hfill & {\frac{2}{{2{\mathrm{cos}}\left( {\sqrt {\mu (f)\varepsilon _{{\mathrm{enz}}}} \frac{\omega }{c}l} \right) - i\left( {\sqrt {\frac{{\varepsilon _{\mathrm{f}}\mu (f)}}{{\varepsilon _{{\mathrm{enz}}}}}} + \sqrt {\frac{{\varepsilon _{{\mathrm{enz}}}}}{{\varepsilon _{\mathrm{f}}\mu (f)}}} } \right){\mathrm{sin}}\left( {\sqrt {\mu (f)\varepsilon _{{\mathrm{enz}}}} \frac{\omega }{c}l} \right)}}} \hfill \end{array}$$

The transmission properties are quantitatively described by Eq. (7), and we report the transmission amplitude and phase in Fig. 1e. As seen, the transmission response of the device is characterized by peak of transmission and zero-phase advance at the operating frequency, in agreement with the theory of EMNZ tunneling. Moreover, the group delay, defined as the negative derivative of transmission phase with respect to the angular frequency, is calculated and reported in Fig. 1f. The group delay of EMNZ tunneling in the lossless case is found to be about 15 times that of a waveguide with same width *W* and filling dielectric *ε*_s_ = 2.65. The dominant physical mechanism of this delay is the resonant character of the dopant, which imposes a time delay before reaching a steady-state field distribution that enables the tunneling effect. Note that this time delay can be controlled as a function of the overall size of the system and the configuration of the dopants. Hence, our proposed scheme could also be employed to effectively slow the light, which are desired in the applications of optical information storage and processing^42^. This effect is preserved even when subjected to moderate losses (<0.5%). Therefore, our theoretical analysis concludes that the scheme of SI photonic doping enables the observation of exotic EMNZ material responses on a single layer substrate processed by the inexpensive PCB technique. It is also noteworthy that, owing to the flexibility of the ENZ material, the proposed method of SI photonic doping holds for the ENZ cavities of arbitrary cross section and even meander style.

### Prototype design and experimental verification

**Fig. 2:**
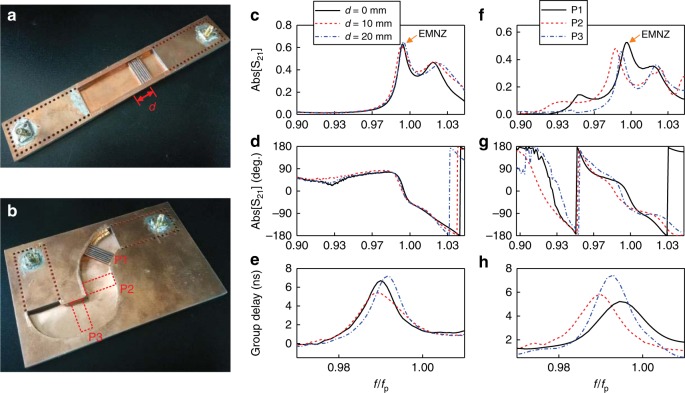
Experimental verification. Fabricated structures for the straight and curved configurations are showed in **a**, **b**. SMA connectors with inner probe of length 4.8 mm are employed to feed the structures. The upper metallic plates of the cavity are not shown in the picture. Transmission amplitudes, phases and group delays of the straight structure with the dopant placed at different distances *d* from the middle of the air cavity are reported in **c**–**e**, respectively. The transmission amplitudes, phases and group delays of the planar curved structure with the dopant placed in different positions P1, P2, and P3, are reported in **f**–**h**, respectively

We provide proof-of-concept demonstrations of SI photonic doping in waveguide structures with straight and curved configurations. The photographs of the fabricated prototypes are shown in Fig. 2a, b. Detailed geometries of the structures are reported on Supplementary Figs. 1 and 2. Both structures are fabricated by the standard PCB technique, using a substrate with a height of 5 mm and a relative permittivity of 2.65. The dielectric dopant is made of ceramic powders with a relative permittivity of 37 and loss tangent of 0.002. Thin metal wires with separation of 1 mm are used to fence the dopant, suppressing the coupling of TM modes in the waveguide, as similarly done in the previous work^38^. The SMA connectors are mounted to stimulate the SIW and receive the transmitted signals. The transmission properties of the two structures doped at different positions are evaluated by vector network analyzer Agilent N9917A (See Methods for the detailed experimental setup). The measured transmission amplitude, phase and group delays of straight structures are reported in the Fig. 2c–e, respectively, and the corresponding results for the curved structures are shown in Fig. 2f–h. We have performed the phase calibration to exclude the phase progress contributed by SIW. The calibrating SIW and its measured transmission phase are presented in Supplementary Fig. 3. A peak of the transmission amplitude near the predesigned frequency *f*_*p*_ accompanied by a near-zero-phase progress and an enhanced group delay is consistently observed in all six measurements, in good agreement with the EMNZ supercoupling theory. In addition, the transmission peaks are verified to be insensitive with respect to changes of the dopant’s position. As the dopant is moved along the cavity of straight-style SI photonic doping structure from *d* = 0 mm, namely the center of the cavity, to *d* = 20 mm, the supercoupling frequency is stably maintained near the prescribed frequency *f*_p_. The small shift of supercoupling peaks for the dopant located in different positions of planar curved structure (Fig. 2f) could be due to the minor variation in the width of the fabricated curved ENZ cavity. The results of full-wave simulation of two SI photonic doping prototypes in the lossless case are gathered in Supplementary Fig. 4. As seen, the measured transmission efficiencies and group delays are lower than the simulated counterparts in the lossless case due to the loss of ceramic powder and the substrate. Decreased transmission amplitude and group delay caused by the dielectric loss of the dopant is simulated and shown in Supplementary Fig. 5. Generally, employing the dopants with smaller loss tangent, reducing the quality factor through using lower permittivity dopant, and/or using gain components would help to reduce the adverse impact of loss, which has to be considered for the realistic scenarios for EMNZ materials. The simulated phase distributions of the magnetic fields are illustrated in Supplementary Figs. 6 and 7, which validate the homogenous field configuration and zero-phase spatial variation over the ENZ cavity. In this manner, our results experimentally and numerically demonstrate both photonic doping position-independence, and geometry-invariant EMNZ supercoupling. Therefore, the proposed scheme of SI photonic doping is a promising candidate to implement the EMNZ response within a planar and integrated design. Based on these results, next sections introduce the design of three different near-zero-index (NZI) devices based on such SI photonic doping.

### Integrated dielectric sensor

**Fig. 3:**
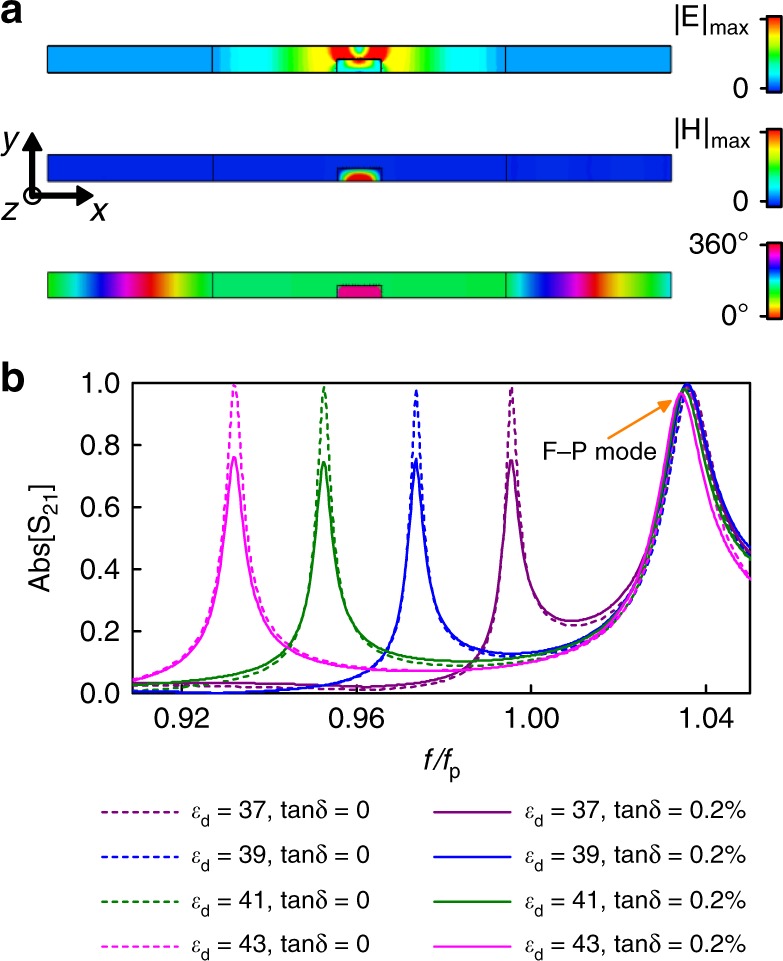
Dielectric sensor based on SI photonic doping. **a** Simulation results at the EMNZ resonance near *f*_*p*_ on the *x–y* cut plane of the straight SI photonic doping structure (see Fig. 1d) for the magnitude of electric field *E*_*y*_ (top), magnitude of magnetic field *H*_*z*_ (middle), and phase of magnetic field *H*_*z*_ (bottom). **b** Transmission amplitudes when using the dopant with different permittivities. The lossless case and lossy dopant with 0.2% dielectric loss tangent are taken into consideration. Dielectric loss tangent (tanδ) is defined by the ratio of the imaginary and real parts of the permittivity

Monitoring of various characters of materials is essential in a wide regime of medical and industrial applications. Here, we provide a compact dielectric sensor of high sensitivity based on the SI photonic doping structure presented in Fig. 3a. The field distributions at EMNZ resonance are carefully examined, with the simulated magnetic field and electric field throughout the structure reported in Fig. 3a. It can be concluded from the figure that the electric energy is concentrated around the edge of the dopant (rigorous proof can be found in Supplementary Note. 2) with about 10 times the magnitude of the incident wave, and the magnitude of magnetic field in the doped region is dramatically strengthened to about 30 times over the incidence, accompanied by a uniform phase over the ENZ cavity. This effect of field concentration and enhancement arises from a combination of the EMNZ resonance and the excitation of strong fields due to the sharp permittivity contrast between the ENZ medium and the dielectric dopant. Intuitively, one can expect that such large field enhancements may lead to high sensitivity to small variations in material parameters. With the help of the SI photonic doping structure provided, we design a dielectric sensor and verify its performance by numerical simulation. As shown in Fig. 3b, a slight variation on dopant’s permittivity (~5%) leads to a noticeable shift of tunneling peak about 0.017 *f*_p_ (110 MHz). That means, a high-resolution measurement of permittivity can be realized by our scheme. The operating frequency range of sensor is also studied, with the frequency of the supercoupling being tuned up to 1.02 *f*_*p*_ when *ε*_d_ = 35 and lower to 0.85 *f*_p_ when *ε*_d_ = 51. The overlap of EMNZ resonance and Fabry-Perot mode at higher frequency region is responsible for the high frequency limit of tuning, while the lowest tuning frequency is determined by the ENZ bandwidth. As also indicated in Fig. 3b, the sensing performance is robust for a moderate loss of the dopant (loss tangent = 0.002). The proposed dielectric sensor based on the EMNZ material empowered by SI photonic doping also features low profile, ease of integration and flexibly, thus providing a potential application in materials characterization, sensing, etc.

### Acousto-microwave modulator

**Fig. 4:**
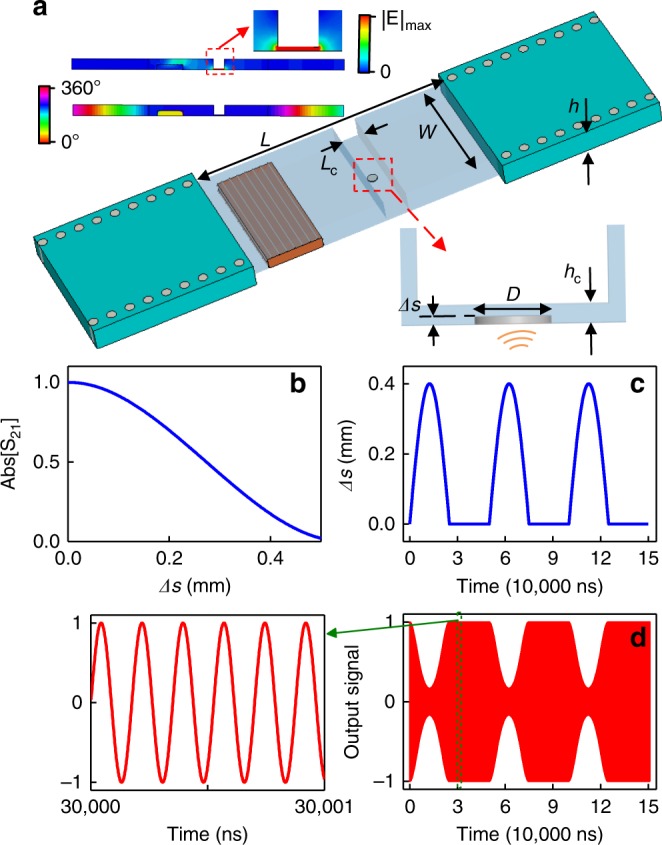
Efficient acoustic modulation of EM Wave based on SI photonic doping. **a** 3D perspective view of our idea for the acoustic modulation structure. The detailed parameters, chosen for our simulation, are *h* = 5 mm, *L* = 64.5 mm, *W* = 25.8 mm. The dopant and the substrate of SIW are the same as those used in the straight SI photonic doping structure (Fig. 1d). Bottom inset: Side view of the channel with *L*_c_ = 5 mm, *h*_c_ = 0.5 mm. A metallic pillar with diameter 2 mm is inserted into this channel with depth Δ*s*. The structure is surrounded by metal. Top inset: Simulated magnitude distribution of the electric field and phase distribution of magnetic field at supercoulping frequency near *f*_p_. **b** The simulated transmission coefficient dependent on the inserted depth of the metallic pillar. **c** Waveform of the modulating signal, i.e., Δ*s* as a function of time. **d** Calculated output modulated signal

We take advantage of the high sensitivity of the EMNZ resonance to propose a novel approach to modulate the electromagnetic wave by weak mechanical vibrations. Our idea of such modulation structure is schematically plotted in Fig. 4a, where we introduce a channel with a thickness of 0.5 mm into the straight SI photonic doping platform. Since EMNZ tunneling is independent of the geometrical deformations of the waveguide, the supercoupling is still supported at the same EMNZ frequency. The mode of operation is verified in the numerical simulations reported in Fig. 4a, which show again a uniform phase distribution of magnetic field within the ENZ cavity, accompanied by near-unity transmission. The electric field is enhanced within the narrow channel (see the inset of Fig. 4a), which implies that even a small obstacle in the channel can significantly influence the transmission rate of supercoupling significantly^42^. Following this intuition, we mount an elastic metallic film on the bottom of the channel, which, for simplicity, is modelled by a metallic pillar with a radius of 1 mm and tunable depth Δ*s* inserted into ENZ cavity. The transmission coefficient at EMNZ resonance versus Δ*s* is simulated and reported in Fig. 4b. As seen, by only a 0.4 mm inserted depth, the transmission coefficient can be reduced from 1 to 0.13. Then, we assume this metallic pillar oscillates with Δ*s* following the function plotted in Fig. 4c: Δ*s*(*t*) = *A*sin(2*πf*_s_*t*) for 2*nπ* < 2*πf*_s_*t* < (2*n* + 1)*π*; Δ*s*(*t*) = 0 for (2*n* + 1)*π* < 2*πf*_s_*t* < (2*n* + 2)*π*, where *n* = 0, 1, 2…, *A* is the amplitude set as 0.4 mm, and *f*_s_ is periodicity of the modulating waveform chosen as 20 KHz, a typical frequency of the acoustic wave. Therefore, by referring to Fig. 4b, c, we can calculate the transmission coefficient varying with time. Finally, the output signal is obtained by considering this amplitude modulation, and the analytical result is plotted in Fig. 4d. As seen, a tiny movement of the pillar or the film driven by the low frequency acoustic wave will take its form clearly in the envelope of the output signal. Therefore, the proposed platform of SI photonic doping offers the opportunity for the acoustic wave or mechanical vibration to efficiently modulate the wave, with its large modulation depth, high sensitivity, and ease of integration. Furthermore, since the frequency of the supercoupling can be flexibly tuned by changing the dielectric constant of the dopant^43^ (see Fig. 3b), the proposed acoustic modulation structure is therefore empowered with the capability of operating at different carrier frequencies, exhibiting the promising potential for reconfigurable devices.

### Electric fiber with flexibility

**Fig. 5:**
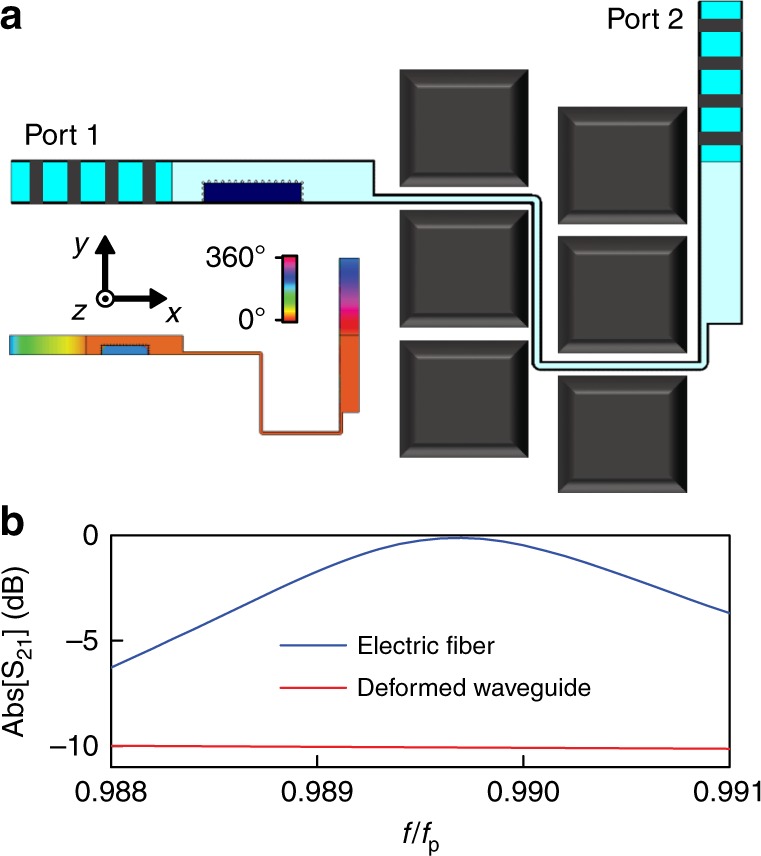
“Electric fiber” based on SI photonic doping. **a** Side view of our idea of the electric fiber, the curved SI photonic doping structure to bypass integrated blocks. The dopant is characterized by relative permittivity *ε*_d_ = 37 and a rectangular cross-section of 12 mm× 2.4 mm. The details of the geometry are reported in Supplementary Fig. 8. Inset: simulation result for the phase of magnetic field distribution *H*_*z*_ on *x–y* cut plane at EMNZ supercoupling near *f*_p_. **b** Transmission amplitudes of the proposed electric fiber and the rectangular waveguide bended into the same configuration

We finish this section by providing a last example of potential application of EMNZ devices based on SI photonic doping: the “electric fiber”, a novel transmission line with high flexibility and arbitrary cross section. Traditional transmission lines, waveguides, and microstrip lines usually require a specific shape and size for their cross-section, and their transmission efficiency can be degraded when exposed to complicated bends. Here, employing the geometry-invariant tunneling of EMNZ material realized by SI photonic doping, we design a new class of flexible transmission lines, named “electric fiber”. The geometry of the configuration is schematically depicted in Fig. 5a, while detailed dimensions are reported in Supplementary Fig. 8. In short, the electric fiber transforms a regular waveguide into a narrow channel to fit in the gaps between blocks and follows the route with sequential right-angle bends. The operation band of electric fiber is set around the EMNZ frequency, characterized by the uniform phase of magnetic field (inset of Fig. 5a). The simulated transmission amplitude of the electric fiber is reported in Fig. 5b, with the expected high transmission rate (near 100%) around EMNZ frequency being verified numerically. A waveguide filled with dielectric of *ε*_s_ = 2.65 is deformed and bended into the same configuration for comparison. As reported in Fig. 5b, such complex bended route and abrupt variation in thickness may deteriorate the performance of the waveguide, with transmission amplitude reduced to −10 dB. The proposed electric fiber based on EMNZ supercoupling also exhibits advantages of efficiency and ease of design and fabrication with respect to ENZ supercoupling^16,17^, since an ENZ channel has to be made, in principle, infinitely thin in order to achieve perfect transmission^16,17^. On the contrary, the geometry of the waveguide is entirely arbitrary for EMNZ supercoupling, allowing us to efficiently use the available space for the layout. In a nutshell, the proposed electric fiber is immune to complicated bends and abrupt change in the cross section, thus being a promising candidate for the transmission of signal on intricate architectures.

## Discussion

The concept of SI photonic doping is proposed to implement EMNZ media into an integrated and planar architecture. To validate the concept, straight and planar curve SI photonic doping structures are prototyped and measured, verifying the properties of position-independent photonic doping and geometry-invariant EMNZ supercoupling. Based on this platform, we proposed three different potential devices: a dielectric sensor, an acousto-microwave modulator, and an electric fiber with flexible geometry. The proposed SI photonic doping represents a new design philosophy, bridging the gap between abstract concept of the near-zero-index material and the applications compatible with the state-of-the-art planar integrated circuits. Our results can be considered as a step forward in the development of NZI technologies with prospective applications in communications, optical/microwave engineering, bio-sensing, and imaging.

## Methods

### Derivation of Eq. (7)

We start from the normalized transfer matrix of a two-port network:

8 $$A = \left[ {\begin{array}{*{20}{c}} {A_{11}} & {A_{12}} \\ {A_{21}} & {A_{22}} \end{array}} \right]$$

Then, the scattering matrix *S* can be derived by the following relationship^32^:

9 $$S = \frac{1}{{A_{11} + A_{12} + A_{21} + A_{22}}}\left[ {\begin{array}{*{20}{c}} {A_{11} + A_{12} - A_{21} - A_{22}} & {2{\mathrm{det}}[A]} \\ 2 & { - A_{11} + A_{12} - A_{21} + A_{22}} \end{array}} \right]$$

Finally, Eq. (7) is obtained by substituting the expression of transfer matrix of the doped ENZ cavity (Eqs. (5) and (6)) into Eq. (9).

### Full-wave simulation

All the numerical simulations were performed using the frequency domain solver of the commercial software CST STUDIO SUITE^®^.

### Experiment setup

The prototypes of SI photonic doping presented in Fig. 2a, b are fabricated on a 5 mm substrate with *ε*_s_ = 2.65 and loss tangent 0.002 by standard printed circuit board (PCB) techniques. The cavities are made through hollowing the predesigned geometries from the substrate, and copper is printed on the top and bottom surfaces of the substrate, as well as on the lateral walls of the cavity. The SMA connector is employed to excite the structures, with its flange soldered with the upper metallic plate of SIW and inner probe of length 4.8 mm inserted in the substrate. The vector network analyzer with 50 Ω coaxial lines connecting the ports evaluates transmission coefficients of the fabricated samples.

## Supplementary information

Supplementary Information

## Data Availability

The simulation and experiment data that support the findings of this study are available from the corresponding author upon reasonable request.
