# Supplementary material for: Substrate-integrated photonic doping for near-zero-index devices
Source: Nat Commun. 2019 Sep 11;10:4132. doi: 10.1038/s41467-019-12083-y (PMC6739333; doi:10.1038/s41467-019-12083-y)
Supplement: Supplementary file 1 — Supplementary Information [file 41467_2019_12083_MOESM1_ESM.pdf]

Substrate-integrated photonic doping for near-zero-index (NZI) devices

Zhou et al.

Supplementary Information

Substrate-integrated photonic doping for near-zero-index (NZI) devices

Ziheng Zhou[1], Yue Li[1,*], Hao Li[1], Wangyu Sun[1], Iñigo Liberal[2], and Nader Engheta[3,*]

[1]Department of Electronic Engineering, Tsinghua University, Beijing 100084, China

[2]Department of Electrical and Electronic Engineering, Public University of Navarre, Pamplona 31006, Spain

[3]Department of Electrical and Systems Engineering, University of Pennsylvania, Philadelphia, PA 19104, USA

**Corresponding author: lyee@tsinghua.edu.cn, engheta@ee.upenn.edu.*

Supplementary Figures

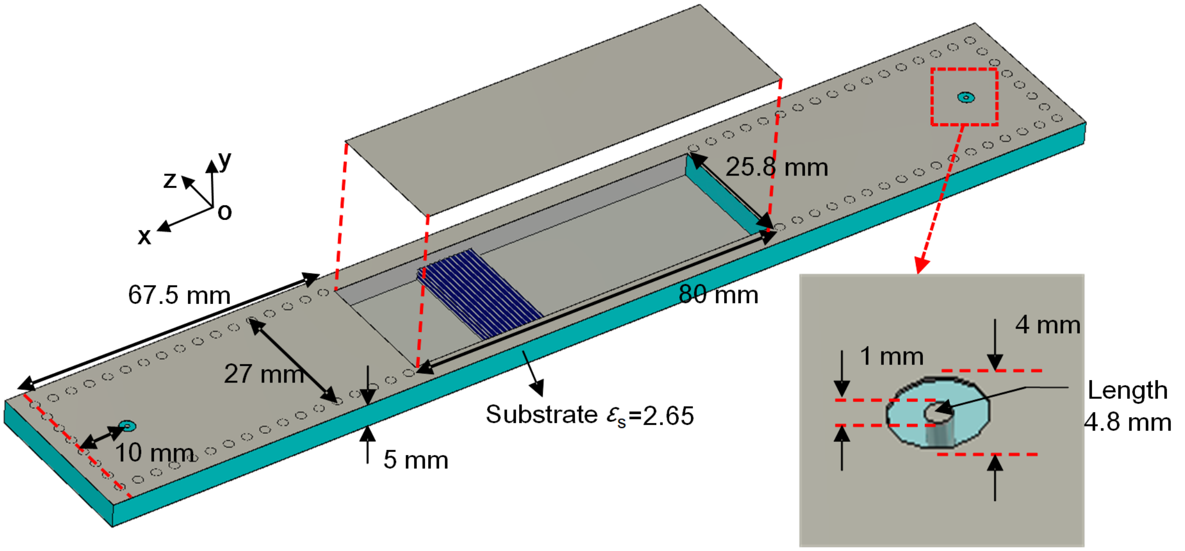

Supplementary Figure 1 | Geometry of the straight SI photonic doping structure. 3D view of the designed SI photonic doping structure with the straight style. Inset: feeding configuration. Parameter values are indicated in the Figure. Metallic vias with radius of 0.5 mm is arranged with the separation of 4 mm. The dopant is characterized by relative permittivity ε_d = 37 and a rectangular cross-section of 12 mm ×2.4 mm on the *x*-*y* plane.

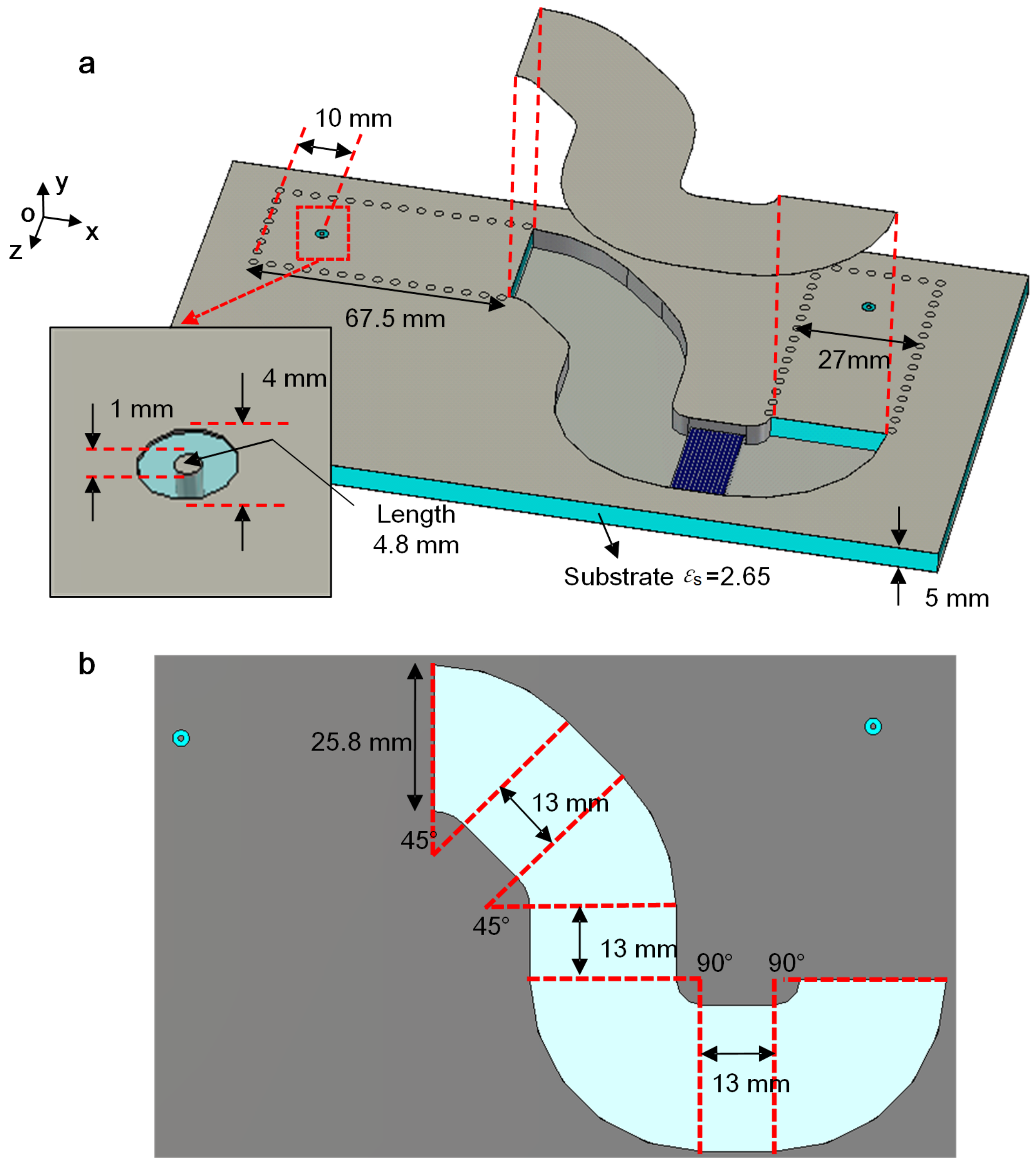

Supplementary Figure 2 | Geometry of the curved SI photonic doping structure. **a** 3D view of the designed SI photonic doping structure with the curved style. Inset: feeding configuration. Parameter values are indicated in the Figure. Metallic vias with radius of 0.5 mm is arranged with the separation of 4 mm. The dopant is characterized by relative permittivity ε_d = 37 and a rectangular cross-section of 12 mm ×2.4 mm on the *x*-*y* plane. **b** Top-view of the curved cavity and description of its geometry.

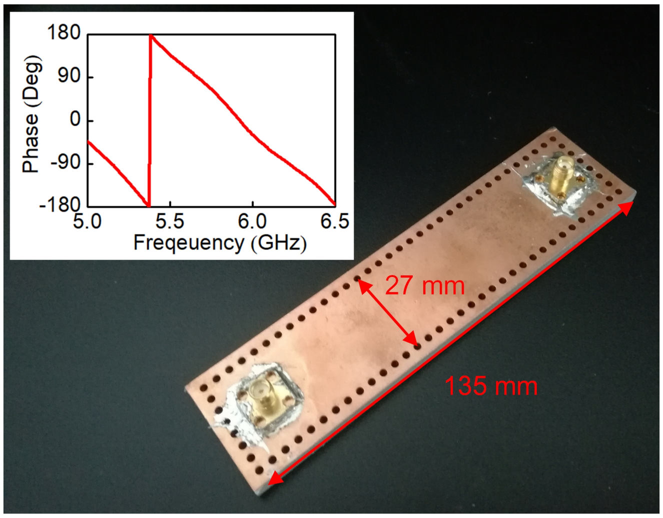

Supplementary Figure 3 | Calibration of the SIW and measurement of the transmission phase. Photograph of the calibration SIW with the same side dimension and the total length as the feeding SIW adopted in Supplementary figures 1 and 2. Metallic vias with radius of 0.5 mm are arranged with the separation of 4 mm. Inset: measured transmission phase as a function of frequency.

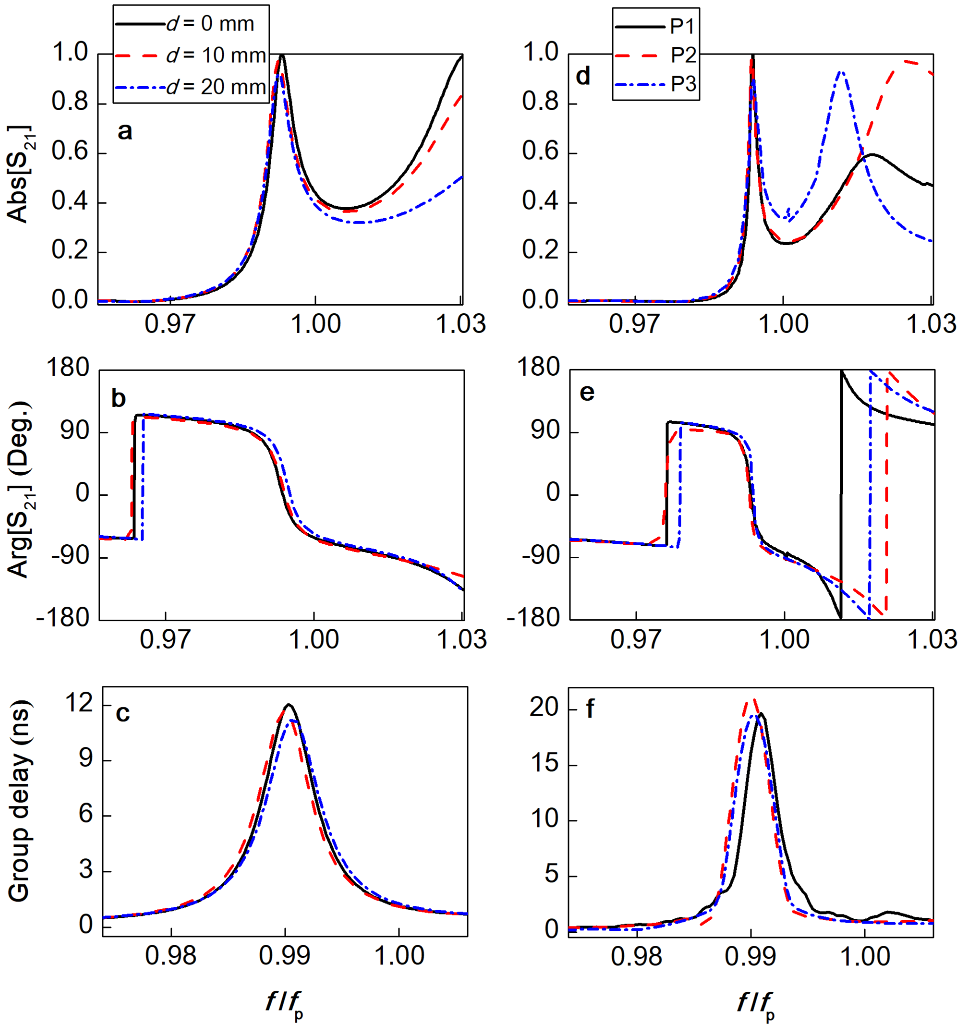

Supplementary Figure 4 | Simulated performance of the SI photonic doping prototypes in the lossless case. Prediction of the transmission amplitudes, phases and group delays for the straight structure with the dopant placed at different distances *d* (refer to Fig. 3 of the main text) away from the middle of the air cavity is reported in **a**, **b** and **c**, respectively. Predicted transmission amplitudes, phases and group delays of the planar curved structure with the dopant placed in different positions P1, P2, and P3 (refer to Fig. 3 of the main text), are reported in **d**, **e** and **f**, respectively. Lossless dopant is characterized by relative permittivity $\varepsilon_d = 37$.

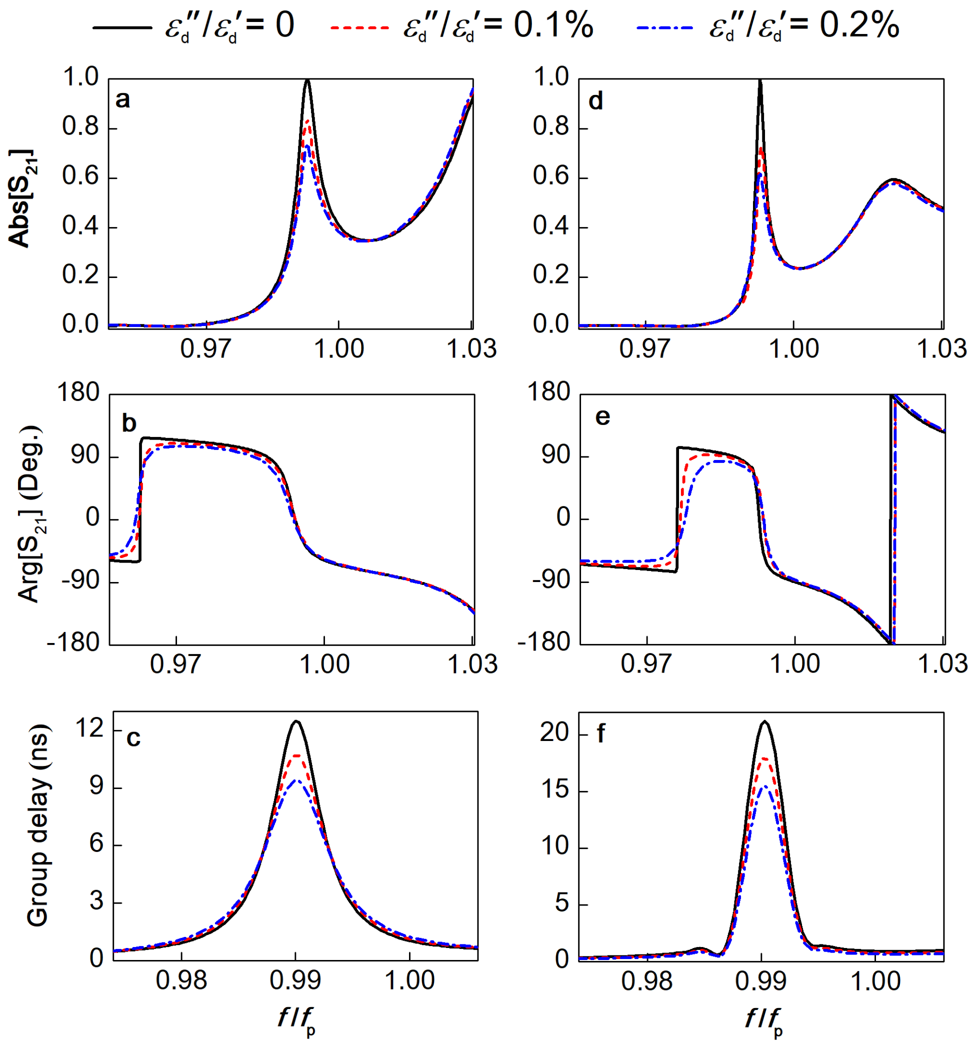

Supplementary Figure 5 | Simulated performance as a function of material loss. ε_d' and ε_d'' represent the real and imaginary part of the permittivity of the dopant, respectively. Predicted transmission amplitudes, phases and group delays of the straight structure with the dopant of different dielectric losses are reported in **a**, **b** and **c**, respectively. The dopant is placed at d =0 mm (refer to Fig.3 of the main text). Predicted transmission amplitudes, phases and group delays of the planar curved structure with the dopant of different dielectric loss are reported in **d**, **e** and **f**, respectively. The dopant is placed in P1 (refer to Fig.3 of the main text).

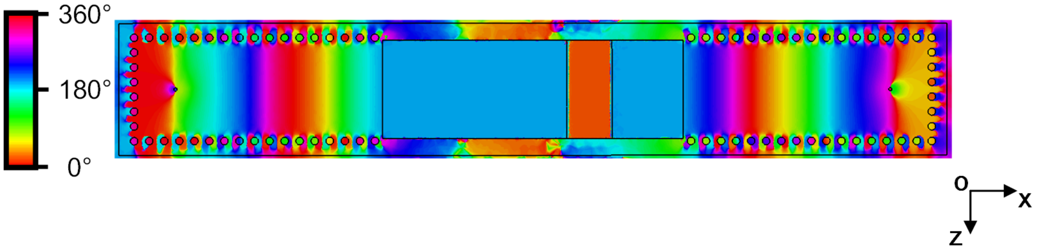

Supplementary Figure 6 | Simulated phase of magnetic field distribution for the straight SI photonic doping structure at the EMNZ frequency. Simulated result for the phase of magnetic field distribution H_z at the EMNZ resonance around f_p on the x-z plane of the straight SI photonic doping structure (see Supplementary figure 1).

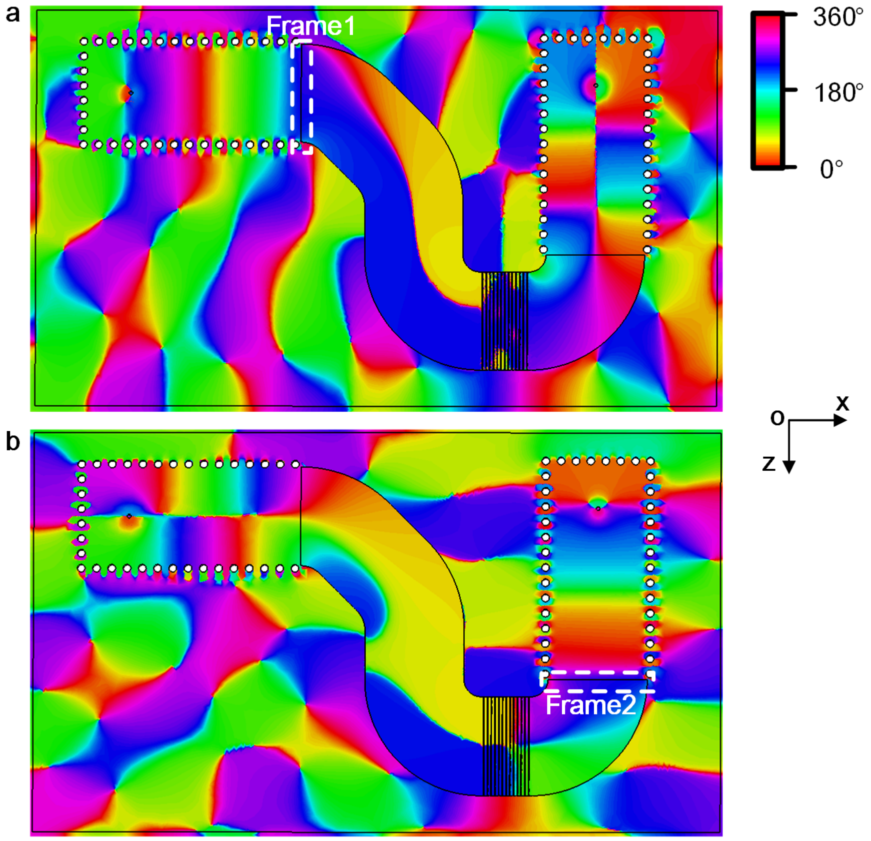

Supplementary Figure 7 | Simulated phase of the magnetic field distribution for the curve SI photonic doping structure at the EMNZ frequency. Simulated result for the phase of magnetic field distribution H_z **a** and H_x **b** at EMNZ resonance around f_p on the x-z plane of the planar curved SI doping structure (see Supplementary figure 2).

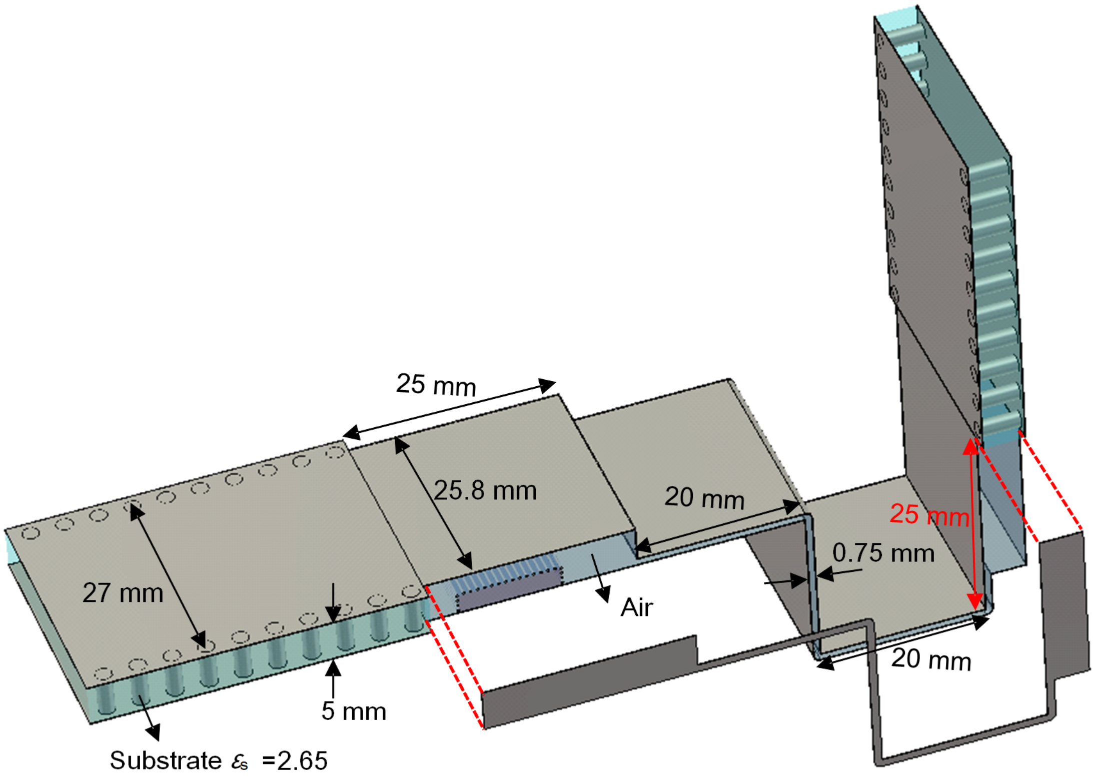

Supplementary Figure 8 | Geometry of proposed electric fiber. 3D view of the designed electric fiber based on SI photonic doping. Parameter values are indicated in the Figure. Metallic vias with radii of 0.5 mm are arranged with the separation of 4 mm. The dopant is characterized by relative permittivity ε_d = 37, and a cross-section of 12 mm × 2.4 mm.

Supplementary Note 1. Green's function technique for solving Equation (1) of the main text

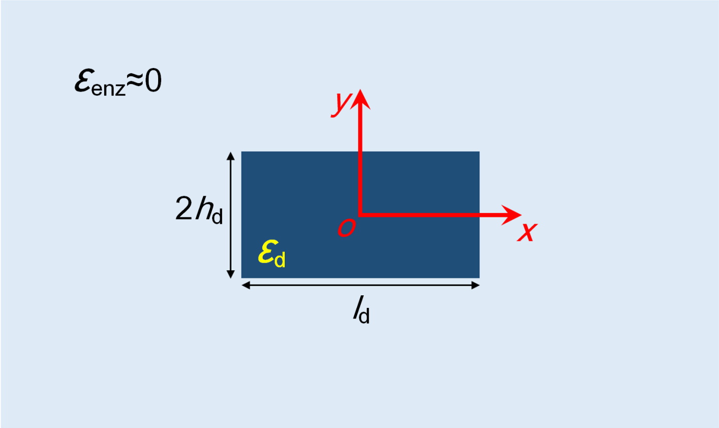

Supplementary Figure 9. Schematic of rectangular dopant in the ENZ medium, the origin of the coordinate is set at the geometrical center of the dopant.

We start by rewriting the partial differential equation and pertaining boundary conditions for the magnetic field distribution within the dopant (see Supplementary Figure 9):

$$\nabla^2\psi(x,y)+k^2\psi(x,y)=0,\quad \psi|_{x=-l_d/2}=\psi|_{x=l_d/2}=\psi|_{y=-h_d}=\psi|_{y=h_d}=1 \tag{S1}$$

, where $k^2=\varepsilon_d\,(\omega/c)^2$. In order to obtain a homogenous boundary condition, we perform the transformation:

$$U(x,y)=\psi(x,y)-1 \tag{S2}$$

Then, (S1) is transformed into:

$$\nabla^2 U+k^2 U=-f(x,y),\quad U|_{x=-l_d/2}=U|_{x=l_d/2}=U|_{y=-h_d}=U|_{y=h_d}=0 \tag{S3}$$

, where $f(x, y) = k^2$ is an excitation term. The Green function of this problem is defined, satisfying the following equation and the boundary condition:

$$\nabla^2 G(x,y;x',y')+k^2 G(x,y;x',y')=-\delta(x-x',y-y'),\quad G|_{x=-l_d/2}=G|_{x=l_d/2}=G|_{y=-h_d}=G|_{y=h_d}=0 \tag{S4}$$

The method of eigenmode expansion[1] is applied to solve the equation (S4), and the solution is obtained as follows:

$$G(x,y;x',y')=\sum_{m=1,n=1}^{+\infty}\frac{U_{m,n}(x,y)U^*_{m,n}(x',y')}{\lambda_{m,n}{}^2-k^2} \tag{S5}$$

, where

$$U_{m,n}(x,y)=\sqrt{2/(h_d l_d)}\cos(m\pi x/l_d)\cos(n\pi y/(2h_d))$$
$$\lambda_{m,n}=\sqrt{(m\pi/l_d)^2+(n\pi/(2h_d))^2} \tag{S6}$$

The subscript *m* and *n* in Eqs. S5 and S6 are odd integers in order to satisfy the boundary condition in Eq. S4.

Finally, with the help of the Green theorem:

$$U(x,y)=\iint_{A_d} G(x,y;x',y')f(x',y')\,\mathrm{d}x'\mathrm{d}y'-\iint_{\partial A_d} U(x',y')\frac{\partial G(x,y;x',y')}{\partial n}\mathrm{d}l' \tag{S7}$$

, the solution of the normalized magnetic field ψ in Eq. (S1) is found to be:

$$\psi(x,y)=U(x,y)+1=1+\sum_{m=1,n=1}^{+\infty}\varepsilon_d\frac{(2\pi f)^2}{c^2}\frac{4((-1)^m-1)((-1)^n-1)}{\pi^2 mn}\frac{\cos(m\pi x/l_d)\cos(n\pi y/(2h_d))}{(m\pi/l_d)^2+(n\pi/(2h_d))^2-\varepsilon_d(2\pi f/c)^2} \tag{S8}$$

The terms with even *n* or *m* vanish in the expression (Eq. S8), hence we can just go over the summation for all the positive integers.

Supplementary Note 2. Theoretical analysis of impact of the waveguide channel length on the EMNZ resonance

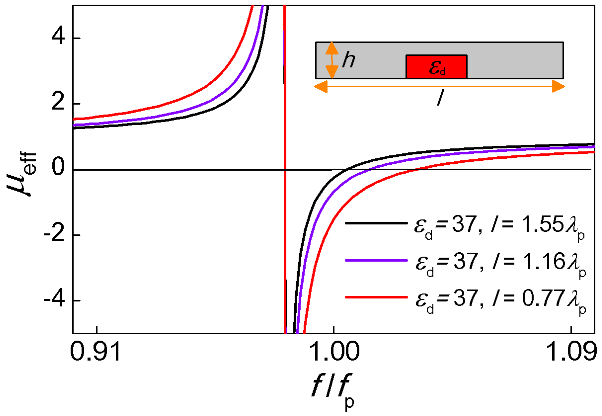

Supplementary Figure 10. Influence of the length of the ENZ channel. The frequency is normalized to f_p = 5.8 GHz. The inset depicts an ENZ channel comprising a dopant.

Here, according to Eq. (4) in the main text, we calculate the effective permeability of the doped ENZ channel with different lengths *l* while keeping the height and the parameter of the dopant the same as those used in the main text. The results are gathered in Supplementary Figure 10. It can be concluded from the figure that reducing the length of

waveguide channel shifts the EMNZ point (zero of effective permeability) to higher frequencies. Intuitively, one can understand that the larger the size of the device, the larger the magnetic flux that must be compensated by the dopant, and, therefore, the closer one has to operate to its resonance to achieve the zero-flux EMNZ condition. To counteract this effect, namely, keep the frequency of EMNZ resonance unchanged, one could simply slightly increase the size or the permittivity of the dopant

Supplementary Note 3. Theoretical analysis of electric field distribution within the dopant at the EMNZ resonance.

Here we check the E-field distribution in the case of the rectangular dopant (referring to Supplementary Figure 9) and prove that the maximum appears at the long sides of the rectangular dopant at the EMNZ resonance. The normalized magnetic field in the dopant has been derived according to our theory (Eq. S8). The electric field is therefore obtained by:

$$\bar{e}(x,y) = (-i\varepsilon_0\varepsilon_{\mathrm{d}}\omega)^{-1}\nabla \times \psi(x,y) \quad \text{(S9)}$$

The calculated magnitude of the electric field at the EMNZ resonance within the dopant is illustrated in Supplementary Figure 11. As seen, the maximum value of the electric field appears at the center of the long side of the rectangular dopant, which is in agreement with the simulation, shown in Supplementary Figure 12. Since the first EMNZ resonance is close to the first PMC point, we have:

$$(m\pi / l_{\mathrm{d}})^2 + (n\pi / (2h_{\mathrm{d}}))^2 \approx \varepsilon_{\mathrm{d}}(\omega / c)^2 \quad \text{(S10)}$$

It is indicated that the first term (m =1, n =1) in the summation of Eq. S8 plays a main role in determining the field distribution, which has the peaks at (x=0, y= ± h_{d}). At the interface of the ENZ medium and the dopant, the continuity of the normal component of the electric displacement vector is imposed. Since the permittivity ENZ medium is infinitely small, the normal electric field in the ENZ medium can be significantly enhanced compared with that at the inner surface of the dopant.

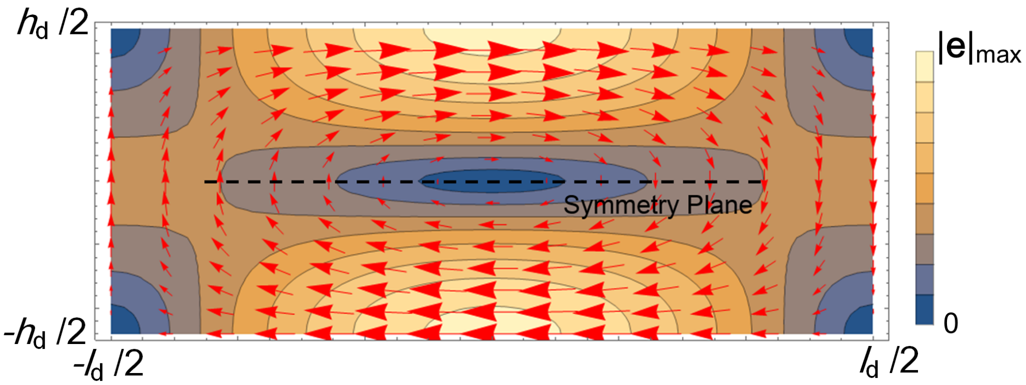

Supplementary Figure 11. Calculated the electric field distribution within the dopant at the EMNZ resonance near f_p.

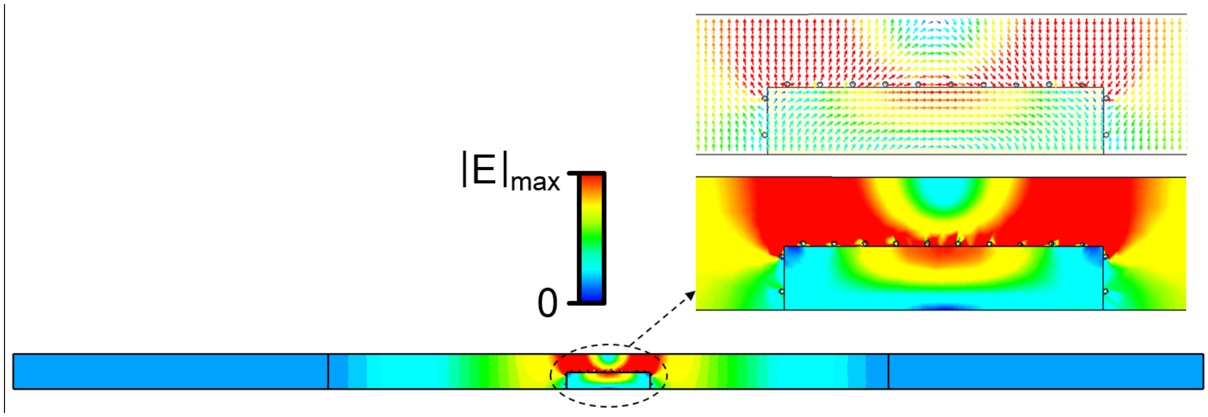

Supplementary Figure 12. Simulated magnitude distribution and vector plot of the electric field within the dopant at the EMNZ resonance near f_p.

Supplementary References

1. Collin, R. E. Field Theory of Guided Waves (Wiley-IEEE Press, 1991).
